# Bleeding Risk in Patients Using Oral Anticoagulants Undergoing Surgical Procedures in Dentistry: A Systematic Review and Meta-Analysis

**DOI:** 10.3389/fphar.2019.00866

**Published:** 2019-08-09

**Authors:** Natália Karol de Andrade, Rogério Heládio Lopes Motta, Cristiane de Cássia Bergamaschi, Luciana Butini Oliveira, Caio Chaves Guimarães, Jimmy de Oliveira Araújo, Luciane Cruz Lopes

**Affiliations:** ^1^Division of Pharmacology, Anesthesiology and Therapeutics, Faculdade São Leopoldo Mandic, Instituto de Pesquisas São Leopoldo Mandic, Campinas, Brazil; ^2^Pharmaceutical Science Graduate Course, University of Sorocaba, Sorocaba, Brazil; ^3^Division of Paediatric Dentistry, Faculdade São Leopoldo Mandic, Instituto de Pesquisas São Leopoldo Mandic, Campinas, Brazil

**Keywords:** oral surgery, oral anticoagulant, bleeding, safety, systematic review, meta-analysis

## Abstract

The management of patients who undergo dental surgical procedures and receive oral anticoagulant therapy requires particular attention due to the risk of bleeding that may occur during the procedure. Bleeding rates in these trans- or post-operative patients tend to be unpredictable. The aim of this study was to conduct a systematic review in order to assess the risk of bleeding during and after performing oral surgery in patients administered oral anticoagulants compared with a group that discontinued anticoagulant therapy. For the purposes of this review, we searched the databases of the Cochrane Central Register of Controlled Trials (CENTRAL), MEDLINE (*via* Ovid), EMBASE (*via* Ovid), and the Virtual Health Library (VHL) from inception of the database to December 2018. The primary outcome was defined as the occurrence of local bleeding during and after oral surgical procedures. Four reviewers, independently and in pairs, screened titles and abstracts for full-text eligibility. Data regarding participant characteristics, interventions, and design and outcomes of the included studies were extracted. The data were pooled using random-effects meta-analyses and described as risk ratios (RRs) with a 95% confidence interval (95% CI). The confidence for the pooled estimates was ascertained through the Grading of Recommendations Assessment, Development, and Evaluation (GRADE) approach, and the protocol of this review was recorded in PROSPERO (CRD42017056986). A total of 58 eligible studies were identified, of which three randomized controlled trials were included in the meta-analysis, covering a total of 323 adult participants, among whom 167 were taking anticoagulants at the time they underwent dental surgery. Of these patients, 14.2% had reported bleeding. The risk of bleeding was found to be one to almost three times greater in patients taking warfarin compared with patients who discontinued the use of anticoagulant during the trans-operative period (RR = 1.67, 95% CI = 0.97 to 2.89) and in the post-operative period (RR = 1.44, 95% CI = 0.71 to 2.92), although the quality of evidence was very low. The results indicate that there is no evidence that the use of anticoagulants eliminates the risk of bleeding during surgical dental procedures.

## Introduction

The routine use of oral anticoagulants is related to hemostatic imbalance between clotting and blood anticoagulation, and significant variations in this relationship may increase the risk of hemorrhage or thromboembolism (Dahlback, 2000; Leiria et al., 2007; Barco et al., 2013; Harter et al., 2015; Yeh et al., 2015; Gerzson et al., 2016).

Anticoagulants can be classified according to their route of administration. Oral anticoagulants include vitamin K antagonists, such as warfarin, that inhibit factors II, VII, IX, and X of the coagulation cascade; new oral anticoagulants that directly inhibit thrombin (coagulation cascade factor II), such as dabigatran; and those that inhibit factor Xa, such as rivaroxaban. A commonly used parenteral anticoagulant is low molecular weight heparin (Barco et al., 2013; Lijfering and Tichellar, 2018).

The increasing use of these medications raises the probability of anticoagulant therapy in patients undergoing dental treatment. Thus, it is necessary to promote safe and preventive treatment in order to avoid complications and comorbidities in these patients (Thacil and Gagg, 2015).

According to the Guidelines of the American College of Chest Physicians (2012), the desirable value of the International Normative Ratio (INR) for patients taking oral anticoagulants is between 2 and 3. When this index indicates anomalies, treatment should be adjusted by the health professional in charge, in order to maintain the patient in a favorable health condition while promoting good short- and long-term prognoses.

Some studies have demonstrated that exodontias performed in patients with a recommendable INR range can be safely conducted without oral anticoagulant interruption or antiplatelet drugs (Campbell et al., 2000; Barrero et al., 2002; Aframian et al., 2007; Morimoto et al., 2008; Goodchild and Donaldson, 2009; Nematullah et al., 2009; Bajkin et al., 2015).

Although previous systematic reviews have assessed the risk of bleeding during oral surgical procedures in patients using oral anticoagulants (Dunn and Turpie, 2003; Madrid and Sanz, 2009; Nematullah et al., 2009; Diermen et al., 2013; Kämmerer et al., 2015; Yang et al., 2016; Shi et al., 2017), the safety of performing dental surgical procedures remains uncertain, on account of the various methodological discrepancies or inconsistencies in these studies. For example, the findings reported by Nematullah et al. (2009) and Madrid et al. (2009) were not restricted to dental surgeries, whereas Diermen et al. (2013) did not perform a meta-analysis of the results, and Kämmerer et al. (2015), Yang et al. (2016), and Shi et al. (2017) used observational studies instead of clinical trials and did not restrict INR values. Furthermore, none of these systematic reviews used GRADE (Grading of Recommendations Assessment, Development and Evaluation), a tool that is routinely used to evaluate the quality of a body of evidence and the strength of recommendations of the findings.

The present review aims to answer the following PICO question: “What is the risk of bleeding in patients who take oral anticoagulants and will undergo oral surgical procedures with or without antithrombotic therapy interruption?” Accordingly, by conducting this systematic review, we aim to determine the risk of bleeding during and after dental surgery in patients undergoing anticoagulant therapy, as well as providing guidance that will assist dental professionals in making informed clinical decisions.

## Methods

### Protocol and Registration

This systematic review was conducted in accordance with the recommendations described in the Cochrane Handbook for Systematic Reviews of Interventions (Moher et al., 2009). The study was performed in accordance with the checklist of PRISMA (Preferred Reporting Items for Systematic Reviews and Meta-Analyses) (Moher et al., 2009; Higgins and Green, 2011). The protocol of this review has been recorded in PROSPERO (International Prospective Register of Systematic Reviews) (protocol CRD42017056986) (http://www.crd.york.ac.uk/PROSPERO) (Motta et al., 2017).

### Eligibility Criteria

#### Study Selection Criteria

Studies considered eligible for inclusion were randomized controlled trials (RCT) involving adult volunteers of both genders, users of oral anticoagulants (vitamin K antagonists, factor Xa inhibitors, or direct thrombin inhibitors), and requiring dental surgeries such as exodontias and dental implants. Included studies were also required to have an experimental group containing participants who were submitted to dental surgeries without interruption of anticoagulant therapy and a control group containing participants who were submitted to dental surgeries with interruption of anticoagulant therapy.

#### Exclusion Criteria

Exclusion criteria included studies in which patients under combined anticoagulant therapy (oral anticoagulant associated with a platelet antiaggregant) represented more than 20% of the sample; studies in which the population was not clearly representative (e.g., patients presenting different bleeding risks due to recent episodes of stroke and recent ablation surgeries); and/or an INR value higher or lower than the desirable range between 2 and 3 in more than 20% of the sample.

#### Outcomes Assessed

Included studies should have reported one of the following outcomes: trans-operative or post-operative local bleeding measured at least 48 h after oral surgical intervention. The definition of bleeding was accepted as described in each study. Oral complications (infections, implant failures, and healing problems at the surgical site) were considered as secondary outcomes.

### Search for Primary Studies

#### Electronic Databases

For the purposes of the review, we performed searches for eligible studies in CENTRAL (Cochrane Central Register of Controlled Trials) part of The Cochrane Library and MEDLINE (*via* Ovid), and EMBASE (*via* Ovid) and VHL (Virtual Health Library) databases from the inception of the database until December 2018, without restrictions relating to language or year of publication. The management of the references (listing and removal of duplicates) was carried out using Endnote X8 software.

#### Other Search Resources

Two reviewers (NA and CB) performed a manual search by reading the reference lists of each selected study or citations found in secondary studies in order to verify potentially eligible studies.

#### Search Strategies

For search purposes, the terms describing the risk of bleeding and oral surgical procedure were combined. The search was conducted using MeSH (Medical Subject Headings) terms for each minor oral surgical procedure (oral surgery, exodontias, and dental implant installation), risk of hemorrhagic events and their synonyms, and several oral anticoagulants (vitamin K antagonists and new oral anticoagulants). The search strategy was adapted for each database and is described in **Supplementary Material A**.

#### Study Selection

Four reviewers (NA and CB; LO and JA), working independently and in pairs (as indicated), selected potentially relevant titles and abstracts, to which the eligibility criteria were applied. The full texts of potentially eligible studies were obtained, and two reviewers (NA and RM) independently assessed the eligibility of each study. All disagreements were resolved through consensus.

#### Data Extraction

Initially, four reviewers (RM and CG; LO and NA), working independently and in pairs, extracted the data from two articles, and a consensus was reached. Discordances were resolved by consensus, and contentious issues were discussed with a third reviewer (LL).

The same reviewers, independently and in pairs, extracted data and recorded information regarding patients, methods, interventions, outcomes, and absence of significant results. A data extraction sheet was used in accordance with the instruction manual prepared by the lead author of this review (NA).

#### Assessment of the Risk of Bias

The Cochrane Collaboration tool was used to evaluate the risk of bias (Altman et al., 1990; Higgins and Green, 2011). All reviewers, independently and in pairs, assessed the risk of bias for each clinical trial according to randomization; allocation concealment; blinding of the patient, the healthcare professional, and the outcome assessors; reporting of incomplete outcomes; and selective reporting of outcomes and imbalance in baseline measurements of the sample.

For each domain, the reviewers assigned response options of “definitely yes,” “probably yes,” “probably not,” or “definitely not,” with “definitely yes” and “probably yes” ultimately indicating a low risk of bias and “definitely not” and “probably not” indicating a high risk of bias (Akl et al., 2013). In cases of disagreement, a consensus was reached through discussion or consultation with a third author (LL).

#### Data Synthesis

Analysis was performed for each anticoagulant and outcome of interest. Confidence in the findings was determined by estimates for each body of evidence. For studies that reported dichotomous outcomes, the relative risk (RR) was calculated combined with a 95% confidence interval (95% CI).

Continuous outcomes were not considered. However, if available they would be described according to the information available in the published protocol of this review (Motta et al., 2017). Details regarding the methods adopted for data synthesis can be found in the same protocol. The random effects associated with meta-analysis were determined using STATA Software (version 10.1).

#### Quality of Evidence

The quality of the evidence was independently assessed (confidence in estimates of the effect) for each outcome reported using the GRADE system (Guyatt et al., 2011a; Guyatt et al., 2011b). In this approach, randomized trials that were initially considered to have a high quality of evidence may have their quality status diminished according to assessment of one or more of the following five categories of limitation: risk of bias (assessed for each study as described previously), inconsistency, indirect evidence, imprecision, and publication bias (Guyatt et al., 2013a, Guyatt et al., 2013b).

The heterogeneity was also evaluated in association with the estimates of the effects using a χ^2^ test and the I^2^ statistic (Higgins and Thompson, 2002) and was classified as follows: 0% to 25% (low heterogeneity), 50% (moderate heterogeneity), and 75% (high heterogeneity) (Higgins and Thompson, 2002).

## Results

### Literature Search Results

**Figure 1:**
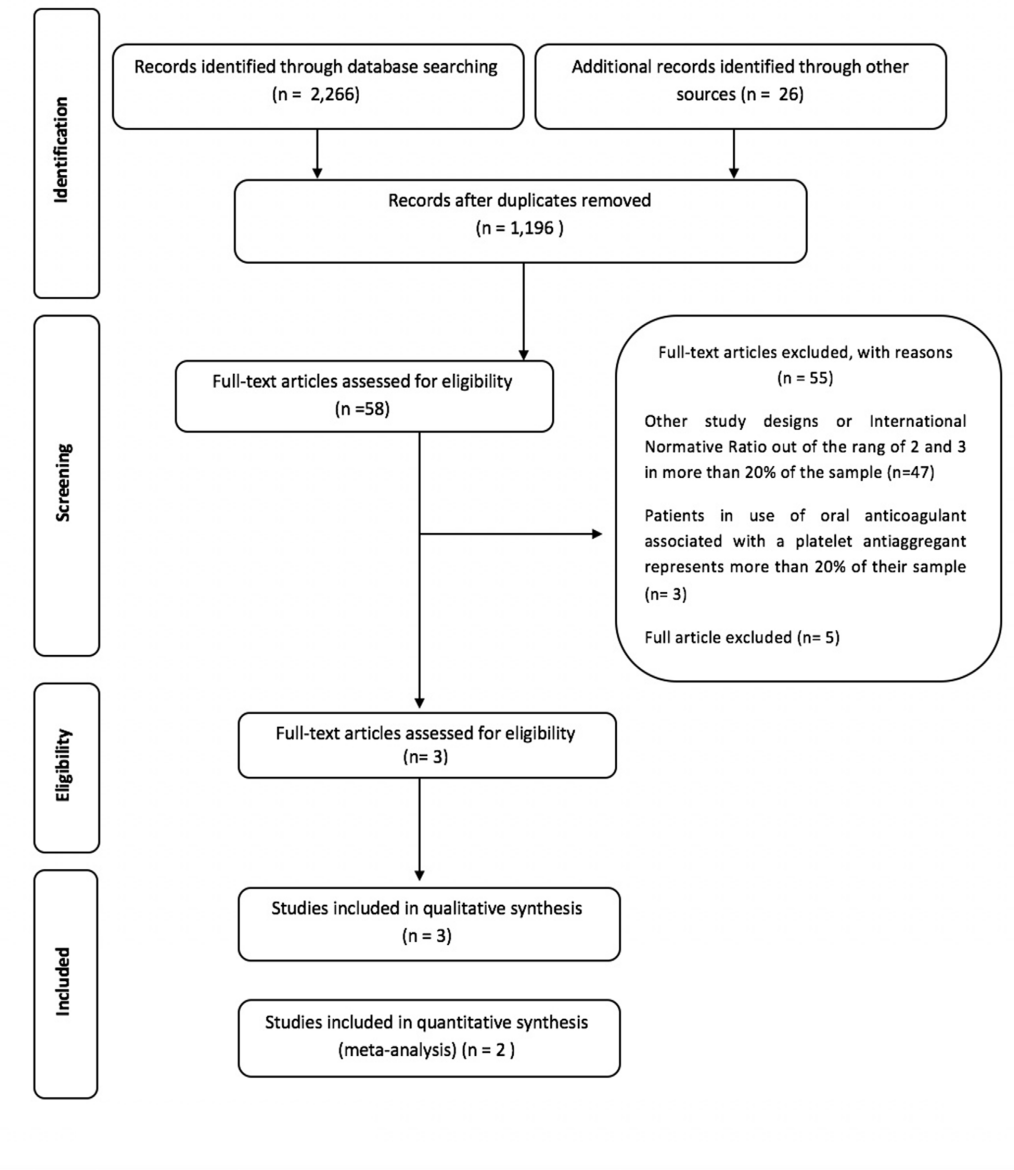
Flow diagram of literature search and selection criteria. Adapted from PRISMA.

From our searches within the aforementioned four databases, we extracted 2,266 publications for consideration, of which 70 articles were duplicates. Accordingly, a total of 2,196 publications were deemed to be potentially eligible. A further 26 publications were identified through manual searches. Of these studies, 58 were considered to fulfil the eligibility criteria established for this review, three of which were included in the qualitative synthesis (Campbell et al., 2000; Evans et al., 2002; Al-Mubarak et al., 2007), and two (Evans et al., 2002; Al-Mubarak et al., 2007) were included in the meta-analysis (**Figure 1**).

### Synthesis Results

The characteristics of the three studies subjected to qualitative synthesis are shown in **Table 1**. These studies evaluated 348 users of oral anticoagulants, most of whom were using vitamin K antagonists. The studies, however, did not present information relating to the underlying diseases that justified the prescription of anticoagulants. All studies compared the risk of bleeding in patients undergoing oral surgical procedures with or without interruption (a few days prior to the dental procedure) of anticoagulant therapy but did not report other outcomes. The list of excluded studies and the reasons for their exclusion are presented in **Supplementary Material B**. Local hemostatic measures, such as compression with gauze and sutures (Campbell et al., 2000; Evans et al., 2002; Al-Mubarak et al., 2007) and sutured oxidized cellulose sponges, were used in the surgical beds in order to control bleeding (Evans et al., 2002).

**Table 1:** Characteristics of included studies.

Study characteristics	Campbell et al. (2000)	Evans et al. (2002)	Al-Mubarak et al. (2007)
Sample (*n* = 348)	25	109	214
Women (%)	NR	33%	67%
Country	USA	United Kingdom	Saudi Arabia
Anticoagulant type	Not specified	Warfarin	Warfarin
Outcome assessed	Bleeding before and after surgery	Bleeding before and after surgery	Bleeding before and after surgery
Follow-up (days)	2	7	7
Surgical procedures (*n* = 345)	22 exodontias (simple or several), 2 alveoloplasties and 1 labial frenectomy	109 exodontias (simple or several)	214 exodontias (simple or several)
Use of hemostatic measures	gauze compression and suture	gauze compression, oxidized cellulose, sponges and suture	Gauze compression and suture
Multicentric study	No	Yes	No
Concomitant drugs	No	Yes (antibiotics)	No
Industry funding	No	No	No

VKA, vitamin K antagonist; INR, International Normative Ratio.

Two of the three studies provided details of patient follow-up during the first week of the post-operative periods. In the third study by Campbell et al. (2000), the results indicated a 2-day follow-up time, although this was not stated explicitly in the text.

The study conducted by Evans et al. (2002) reported the concomitant use of other drugs during the research period, in which some patients received antibiotic prophylaxis. None of the studies received industrial funding. Although all three studies reported the primary outcome “local bleeding,” none reported secondary outcomes.

Campbell et al. (2000) performed a qualitative evaluation of the bleeding associated with minor oral surgeries in patients with VKA (the study does not specify the anticoagulant). Bleeding was assessed in terms of “surgical sponge weight” in the pre- and post-operative periods. In this regard, it was considered that each gram of blood is equivalent to a volume of 1 ml, and accordingly the volume of blood lost in the pre- and post-operative periods was determined from the difference in sponge weight. Twenty-five patients were allocated to one of two groups (mean INR of 2.0 ± 0.5): the “experimental group” (*n* = 12) in which patients’ use of anticoagulants before and after surgical procedures was maintained, and the “control group” (*n* = 13) in which anticoagulant use was interrupted 72 to 96 h before the surgical intervention. None of the patients presented trans- or post-operative bleeding requiring any therapeutic intervention, and there was no significant difference in blood loss between the groups. Although the authors have suggested the need for additional investigations, they also consider that it would be possible to perform minor oral surgical procedures in patients taking oral anticoagulants without additional pharmacological interventions.

Evans et al. (2002) evaluated the risk of bleeding in 109 individuals taking warfarin, who underwent simple and multiple exodontia. Bleeding was assessed by the use of gauze, which the patient was instructed to bite on for a period of 10 min. If the hemostatic measures were not sufficient to contain the bleeding, the event was considered as “immediate bleeding.” Patients were randomly assigned to one of the two study groups by allocation concealment (57 to the experimental group and 52 to the control group). Individuals in the experimental group continued using warfarin, whereas those in the control group interrupted the use of warfarin 2 days prior to the surgical procedure. All surgical beds were covered with oxidized cellulose sponges, and the patients were sutured. The patients were instructed to bite a gauze pad for 10 min. If such local hemostatic measures were not sufficient to contain bleeding, it was considered “immediate bleeding.” Twenty-two patients presented complications related to bleeding (post-surgery only), 15 (26%) of whom were in the experimental group and seven (13.5%) (*n* = 7) were in the control group. The study thus indicates the possible heightened risk of complications associated with bleeding when warfarin is maintained. In most cases, however, these events can be controlled by administering local treatment.

Al-Mubarak et al. (2007) evaluated the incidence of post-operative hemorrhage in patients taking warfarin (dose of 2 to 10 mg daily) undergoing exodontia. The bleeding was assessed by the use of gauze on which the patient was instructed to bite for a period of 6 to 10 min. If these hemostatic measures were not sufficient to contain the bleeding, the event was considered as bleeding. Additionally, the volunteers were followed for up to 7 days in order to verify the presence of hemorrhagic sites (evaluated in terms clot formation and the local repair process). A total of 214 patients were randomly divided into four groups. Patients in groups 1 and 3 interrupted warfarin intake (2 days before the surgical procedure): group 1 (*n* = 48, mean INR 1.8 and no suture) and group 3 (*n* = 56, mean INR 1.9 and with suture). Patients in groups 2 and 4 maintained warfarin use: group 2 (*n* = 58, mean INR 2.4 and without suture) and group 4 (*n* = 52, mean INR 2.7 and with suture). All patients received gauze compression for 6 to 10 min and were followed for up to 7 days. It was observed that among the patients in groups 1 and 2, 12% (*n* = 6) and 21% (*n* = 12) presented trans-operative hemorrhagic events, respectively. During the post-operative period, 4% (*n* = 2) of the patients in group 1 and 3% (*n* = 2) in group 2 presented bleeding.

### Risk of Bias Assessment

**Figure 2:**
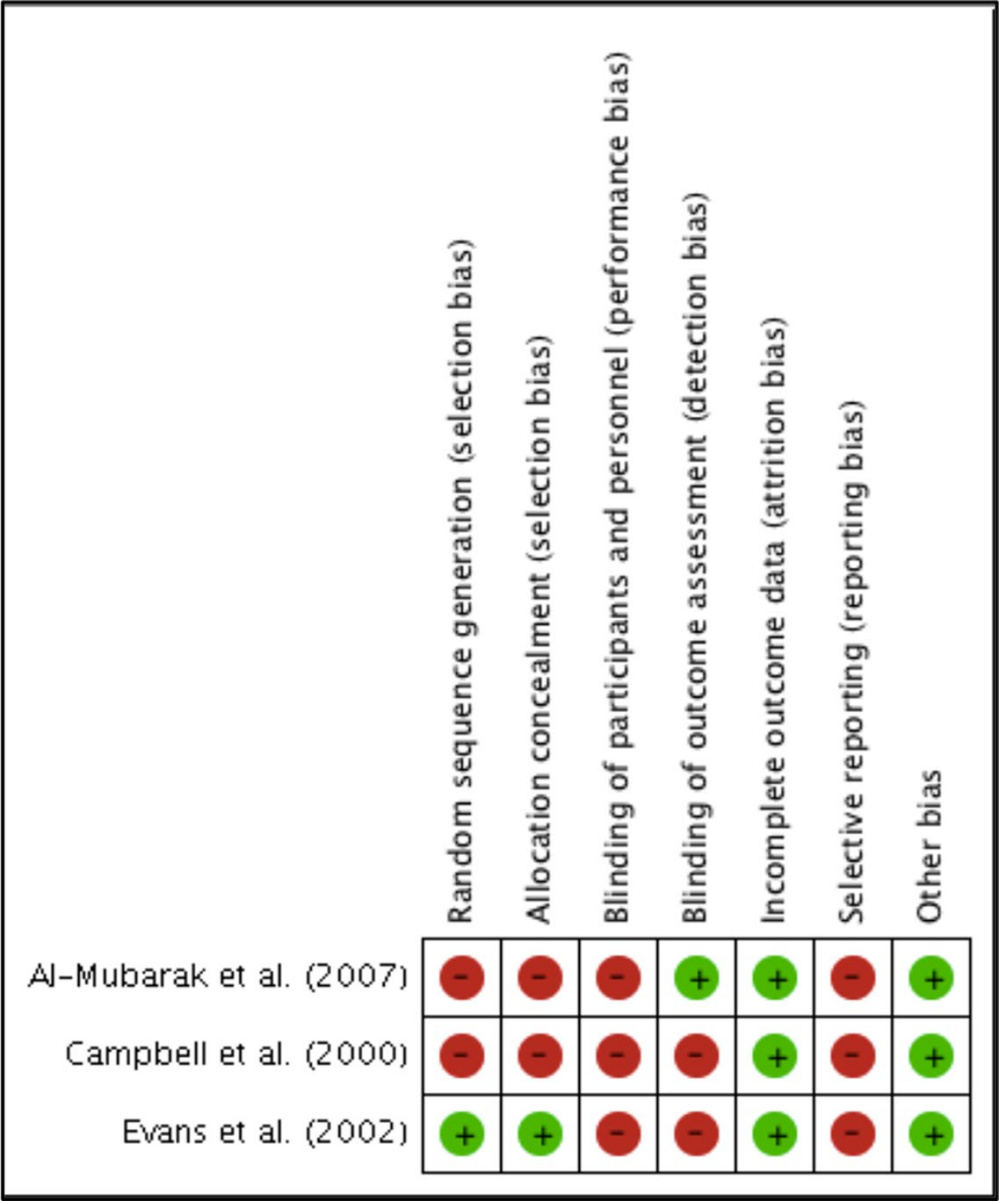
Authors’ consensus on the risk of bias of each included study.

**Figure 3:**
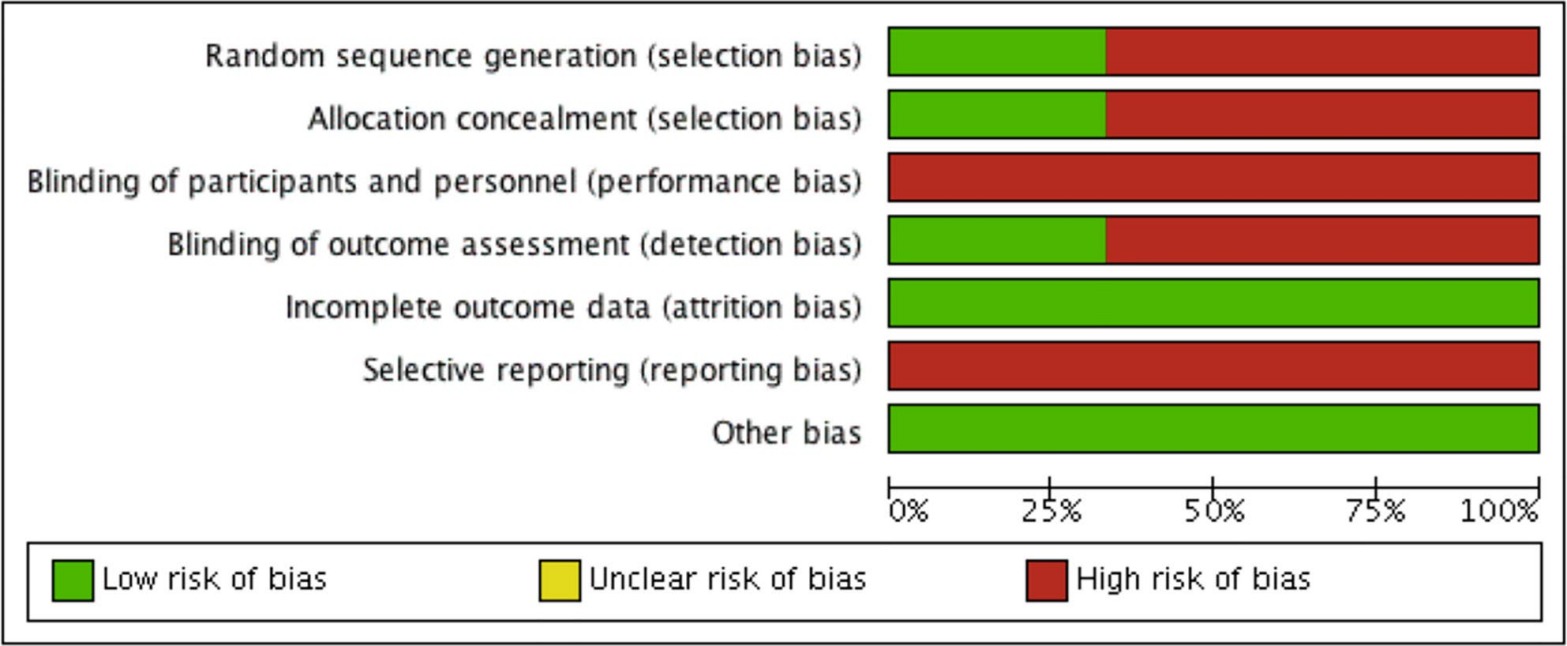
Risk of bias of included studies.

As shown in **Figures 2** and **3**, the three included studies had a high risk bias. However, the three studies did not provide sufficient data regarding the randomization process to enable an evaluation of potential selection bias (Campbell et al., 2000; Al-Mubarak et al., 2007). Evans et al. (2002), however, describe the generation of random sequences of patients in the groups.

In the studies reported by Campbell et al. (2000) and Al-Mubarak et al. (2007), concealment of the allocation was not guaranteed, and none of the studies provided sufficient information in order for us to determine whether there was blinding of the patients or of those involved in the research, which may have caused detection bias, as the outcomes evaluated are likely to be influenced by the absence of blinding. Only Al-Mubarak et al. (2007) provided an indication that there was blinding of the professionals who evaluated the outcomes, although they did not clearly describe how the procedure was performed.

Furthermore, none of the studies clarified whether there was follow-up loss, although all included studies described the evaluated outcomes. However, apparently, all patients enrolled in the studies initiated and completed the proposed treatments.

The incidence of thromboembolic events among patients who interrupted anticoagulant therapy was not assessed in clinical trials. Other secondary outcomes, such as bruising, ecchymoses, or post-operative infections, may have been considered by the studies; however, these were not reported. None of the studies presented the protocol record, which would have enabled us to infer the risk of uncertain bias.

None of the included studies obtained industrial funding, and based on a reading of the studies, no other problems were identified in addition to those already mentioned; thus, we assume that the studies were free of other potential sources of bias.

### Results of Evaluated Outcome and Quality of Evidence

**Figure 4:**
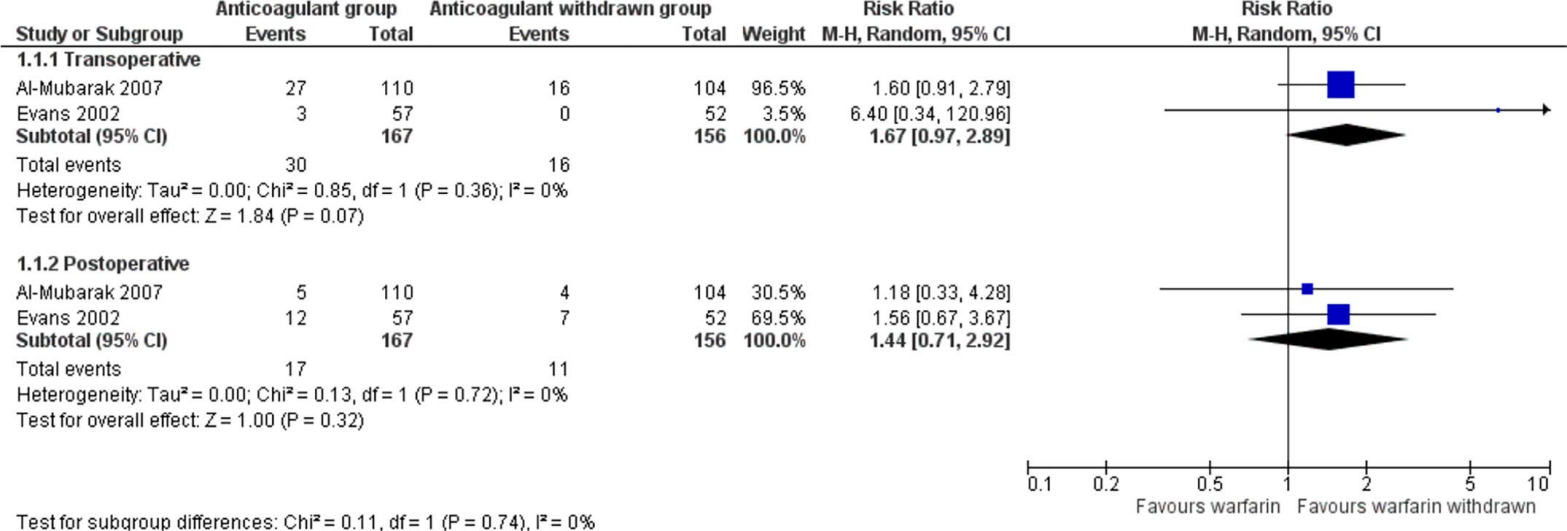
Meta-analysis of the risk of bleeding in patients using warfarin in the trans- and post-operative periods.

Random-effects meta-analysis revealed no statistically significant difference between the groups that continued or interrupted the use of anticoagulants. Nevertheless, the results have demonstrated a one to almost three times greater bleeding risk in patients taking warfarin compared with patients who discontinued the use of anticoagulant in the trans-operative (RR = 1.67, 95% CI = 0.97 to 2.89) and post-operative (RR = 1.44, 95% CI = 0.71 to 2.92) periods (**Figure 4**). Sub-group analyses were not possible due to the low number of studies included.

The quality of evidence according to GRADE (**Table 2**) concerning “bleeding risk” (the main outcome) in both trans- and post-operative periods was considered very low, which indicates a very small confidence in the estimated effect. Furthermore, we also noted that the studies were associated with a high risk of bias, and thus the relative estimate of results may not be reliable. We did, however, observe an overlap in the confidence intervals of the studies, and therefore, the inconsistency was considered unimportant. Moreover, low heterogeneity was observed between the results.

**Table 2:** Quality of study evidence according to Grading of Recommendations Assessment, Development, and Evaluation (GRADE).

Measured parameters							Number of patients		Effect		Quality	Outcome importance/relevance
# of studies	Study type	Risk of bias	Inconsistency	Indirectness evidence	Imprecision	Other considerations	Used oral anticoagulants	Did not use oral anticoagulants	Relative risk (95% CI)	Absolute risk (95% CI)		
Bleeding risk (transoperative)												
2	Randomized clinical trials	Very severe^a^	Not severe	Not severe	Severe^b^	None	30/167 (18.0%)	16/156 (10.3%)	1.67 (0.97 to 2.89)	69/1,000 (3 to 194)	⨁◯◯◯very low	CRITICAL
Bleeding risk (postoperative)												
2	Randomized clinical trials	Very severe^a^	Not severe	Not severe	Severe^b^	None	17/167 (10.2%)	11/156 (7.1%)	1.44 (0.71 to 2.92)	31/1,000 (20 to 135)	⨁◯◯◯very low	CRITICAL

RR, relative risk; 95% CI, 95% confidence interval. ^a^Al-Mubarack’s study presented a risk of bias for randomization, concealment of allocation and blinding and the Evans’ study presented problems in blinding. ^b^Number of total events is small and the confidence interval is wide with no effect.

The included studies presented details of interventions and outcomes of interest, and therefore the evidence was considered direct. Considerable imprecision was noted with regard to the number of total events and the small number of samples, in addition to the wide confidence intervals with no true effect.

Although each of the included studies reported few bleeding events, the lack of any details on other meaningful outcomes, including the incidence of thromboembolism or local complications, limits the scope of the clinical decision making, such as the interruption or maintenance of antithrombotic therapy prior to dental surgical procedures. The absence of such details may suggest a publication bias.

## Discussion

Excessive trans-or post-operative bleeding is a negative outcome in dentistry that limits several surgical interventions, and in this regard, an evaluation of the risk of bleeding in users of oral anticoagulants may contribute to minimizing the complications experienced by dental patients.

In this study, we evaluated the risk of bleeding in patients using oral anticoagulants who underwent dental surgical procedures. For the purposes of the descriptive analysis, we performed a detailed assessment of three relevant studies (Campbell et al., 2000; Evans et al., 2002; Al-Mubarak et al., 2007), two of which were included in the meta-analysis (Evans et al., 2002; Al-Mubarak et al., 2007).

The assessments undertaken in the present review are restricted to warfarin, for which no recent RCTs have been published. Although our meta-analysis indicates an increased risk of bleeding when patients maintain the use of this anticoagulant, the findings of the assessed studies present very poor-quality evidence, which represents a constraint in terms of making any definite recommendations.

It is noteworthy, however, that apart from bleeding, no other important relevant outcomes, such as ecchymoses and thromboembolism, were reported in the trials included in this review and therefore could not be evaluated. Further, we were unable to perform sub-group analyses, on account of the small number of studies included. Given that the frequency of thromboembolic events is an important outcome for assessing safe antithrombotic therapy interruption, the absence of any report on this outcome in clinical trials may suggest bias due to the selective reporting of outcomes.

According to the guidelines of the American College of Chest Physicians Holbrook et al. (2012), a safe INR for dental interventions ranges from 2 to 3. This INR range has not always been considered as an eligibility criterion in previous studies, as comparisons are difficult and a larger amplitude of INR becomes an inconclusive factor, particularly for those patients who are users of vitamin K antagonists. In the present study we used patient INR values ranging from 2 to 3 as an inclusion criterion for clinical trials, which necessarily limited the number of studies we were able to select for the review. In addition to the INR interval adopted by the studies, other disparities were observed in the systematic reviews previously published on this subject (Dunn and Turpie, 2003; Madrid and Sanz, 2009; Diermen et al., 2013; Kämmerer et al., 2015; Yang et al., 2016; Shi et al., 2017), which accordingly highlight the relevance of the present study.

In a systematic review conducted by Dunn and Turpie (2003), the authors identified a low incidence of thromboembolic events in patients who had discontinued anticoagulant therapy (1.6% of patients). However, this finding was based on a consideration of both medical and oral procedures. Moreover, the authors of this review did not assess the risk of bias or the quality of the evidence of the findings.

Some systematic reviews have indicated that there is no difference in the risk of bleeding among patients who interrupt and those who maintain anticoagulant therapy prior to undergoing surgical procedures (Madri and Sanz, 2009; Yang et al., 2016), which corroborates the findings of the present study. However, the findings of Kämmerer et al. (2015) and Shi et al. (2017) have indicated an increased risk of bleeding in patients who maintain anticoagulant therapy during the period in which they undergo dental procedures. Nevertheless, they concluded that such complications can be readily treated using local hemostatic measures. However, none of these studies included only RCTs, and none took INR values into account.

Kämmerer et al. (2015) emphasized that the risk of potentially fatal thromboembolism due to interruption of anticoagulant therapy outweighs the risk of post-operative bleeding episodes. The authors suggest that minor procedures, such as exodontias and dental implants, can be safely performed if the INR is within the therapeutic range and local hemostatic measures are used.

Diermen et al. (2013) evaluated the level of evidence and grade of recommendation (regardless of study design and including clinical practice guidelines) of studies that examined the risk of discontinuing treatment with antiplatelet agents and anticoagulants prior to oral surgical procedures. The results indicated that antithrombotic therapy should not be interrupted for simple dental procedures.

Although the review performed by Yang et al. (2016) also included the studies of Evans et al. (2002) and Al-Mubarak et al. (2007), it was essentially a qualitative analysis and considered other study designs in addition to RCTs. The authors suggested that patients who maintain oral anticoagulant therapy are not at any greater risk of bleeding after dental extractions than are patients who have discontinued oral anticoagulant therapy.

### Strengths and Limitations of This Study

The present study was carried out with methodological accuracy, including an evaluation of the risk of bias and an assessment of the quality of evidence, which have not featured in the previously published systematic reviews of this topic (Dunn and Turpie, 2003; Madrid and Sanz, 2009; Nematullah et al., 2009; Diermen et al., 2013; Kämmerer et al., 2015; Yang et al., 2016; Shi et al., 2017). Thus, we have explicitly highlighted the eligibility criteria, the comprehensive database search, and the independent and paired evaluation of each study. Moreover, the use of GRADE made it possible to evaluate the strength and the quality of the body of evidence in relation to the effect of bleeding risk.

The specific criteria used for the selection of included studies do, however, represent a limiting factor for the findings of this review, owing to our requirement regarding the methodological quality of the RCT. This, nevertheless, highlights the necessity for a greater number of primary studies on this subject, with greater methodological accuracy in order to increase the reliability of the findings.

### Implications for Clinical Practice and Future Research

Our findings have revealed that there is no statistical difference in the risk of bleeding in warfarin users undergoing dental surgery without anticoagulant therapy interruption compared with those who discontinue the therapy. However, due to the poor quality evidence, the most appropriate practice with respect to whether oral anticoagulant therapy should be interrupted remains uncertain. Meticulous methodological quality should be encouraged with regard to answering the question posed at the outset, in addition to emphasizing the use of INR as a standardized criterion for future research.

On the basis of our findings, we recommend dental surgical planning based on a diagnosis of the general health condition of the patient. This diagnosis implies the accomplishment of a comprehensive anamnesis, including the diseases involved and use of the drugs related to blood hemostasis. This evaluation should additionally include the request for a complete blood exam and determine whether the patient is decompensated or presents comorbidities that contribute to a greater risk associated with the surgical procedures. Moreover, it is important that they be referred to the doctor in charge for a more specific opinion and classification of risk.

To date there have been no RCTs that have evaluated the outcomes of interest of this research in relation to new oral anticoagulants. Given this scenario, new studies with variable control and the use of standardized methods for determining outcomes should be developed.

## Conclusion

The findings of this analysis indicate that there is no evidence of a greater risk of bleeding in patients using oral anticoagulants who undergo surgical dental procedures. However, the findings should be interpreted with caution and new studies on the subject should be initiated.

## Author Contributions

NA is the main investigator and led the development and the writing of the manuscript. LL, RM, and CB are the project managers and co-investigators, who contributed to the development, writing, and revision of the manuscript. LO, CG, and JA are co-investigators and contributed to the development and revision of the manuscript. All authors have read and approved the final manuscript.

## Conflict of Interest Statement

The authors declare that the research was conducted in the absence of any commercial or financial relationships that could be construed as a potential conflict of interest.
