# Supplementary material for: Bleeding Risk in Patients Using Oral Anticoagulants Undergoing Surgical Procedures in Dentistry: A Systematic Review and Meta-Analysis
Source: Front Pharmacol. 2019 Aug 9;10:866. doi: 10.3389/fphar.2019.00866 (PMC6696826; doi:10.3389/fphar.2019.00866)
Supplement: Supplementary file 1 [file Table_1.docx]

**Supplementary material A:** Search strategy of MEDLINE (via Ovid) database

| 1 | Oral surgery |
| --- | --- |
| 2 | Maxillofacial Surgery |
| 3 | Surgery, Maxillofacial |
| 4 | Exodontics |
| 5 | Dentistry, Operative |
| 6 | Oral Surgical Procedures. |
| 7 | Dental Implants |
| 8 | Implants, Dental |
| 9 | Dental Prostheses, Surgical |
| 10 | Dental Prosthesis, Surgical |
| 11 | Surgical Dental Prostheses |
| 12 | Surgical Dental Prosthesis.mp. |
| 13 | Prostheses, Surgical Dental |
| 14 | Prosthesis, Surgical Dental |
| 15 | #1 or #2 or #3 or #4 or #5 or #6 or #7 or #8 or #9 or #10 or #11 or #12 or #13 or #14 |
| 16 | bleeding |
| 17 | bleed |
| 18 | microbleed |
| 19 | risk |
| 20 | risk factor |
| 21 | risk assessment |
| 22 | #16 or #17 or #18 or #19 or #20 or #21 |
| 23 | coumarin derivative |
| 24 | warfarin |
| 25 | heparin |
| 26 | nadroparin |
| 27 | clexane |
| 28 | lovenox |
| 29 | dalteparin |
| 30 | ardeparin |
| 31 | tinzaparin |
| 32 | logiparin |
| 33 | certoparin |
| 34 | sandoparin |
| 35 | reviparin |
| 36 | danaproid |
| 37 | bemiparin |
| 38 | semuloparin |
| 39 | trometamol |
| 40 | parnaparin |
| 41 | fluxum |
| 42 | #23 or #24 or #25 or #26 or #27 or #28 or #29 or #30 or #31 or #32 or #33 or #34 or #35 or #36 or #37 or #38 or #39 or #40 or #41 |
| 43 | #15 and #22 and #42 |

Supplementary material B: Reasons for exclusions

| Clinical trial no randomized | 1. Salam S, Yusuf H, Milosevic A. [Bleeding after dental extractions in patients taking warfarin]. Br J Oral Maxillofac Surg. 2007 Sep; 45(6):463-6. 2. Rocha AL, Souza AF, Martins MAP, Fraga MG, Travassos DV, Oliveira ACB, et al. [Oral surgery in patients under antithrombotic therapy: perioperative bleeding as a significant risk factor for postoperative hemorrhage]. Blood Coagul Fibrinolysis. 2018 Jan; 29(1):97-103. 3. Hong CH, Napeñas JJ, Brennan MT, Furney SL, Lockhart PB. Frequency of bleeding following invasive dental procedures in patients on low-molecular-weight heparin therapy. J Oral Maxillofac Surg. 2010 May; 68(5):975-9 4. Eichhorn W, Burkert J, Vorwig O, Blessmann M, Cachovan G, Zeuch J, et al. [Bleeding incidence after oral surgery with continued oral anticoagulation]. Clin Oral Investig. 2012 Oct;16(5):1371-6. 5. Ferrieri GB, Castiglioni S, Carmagnola D, Cargnel M, Strohmenger L, Abati S. Oral surgery in patients on anticoagulant treatment without therapy interruption. J Oral Maxillofac Surg. 2007;65(6):1149-1154. 6. Morimoto Y, Niwa H, Hanatani A, Nakatani T. [Hemostatic management during oral surgery in patients with a left-ventricular assist system undergoing high-level anticoagulant therapy: efficacy of low molecular weight heparin]. J Oral Maxillofac Surg. 2008 Mar;66(3):568-71. 7. Cieślik-Bielewska A, Pelc R, Cieślik T. [Oral surgery procedures in patients on anticoagulants. Preliminary report.] Kardiol Pol. 2005 Aug;63(2):137-40. 8. Kataoka T, Hoshi K, Ando T. [Is the HAS-BLED score useful in predicting post-extraction bleeding in patients taking warfarin? A retrospective cohort study.] BMJ Open. 2016 Mar 2;6(3):e010471. 9. Hanken H, Gröbe A, Heiland M, Smeets R, Kluwe L, Wikner J, et al. [Postoperative bleeding risk for oral surgery under continued rivaroxaban anticoagulant therapy.] Clin Oral Investig. 2016 Jul;20(6):1279-82. 10. Gröbe A, Fraederich M, Smeets R, Heiland M, Kluwe L, Zeuch J, et al. [Postoperative bleeding risk for oral surgery under continued clopidogrel antiplatelet therapy]. Biomed Res Int. 2015;2015:823651. | |
| --- | --- | --- |
| Literature review | 1. Aframian, DJ, Lalla RV, Peterson DE. [Management of dental patients taking common hemostasis-altering medications]. Oral Surg Oral Med Oral Pathol Oral Radiol Endod. 2007;103 Suppl:S45–S11. 2. Balevi B. [Should warfarin be discontinued before a dental extraction? A decision-tree analysis.] Oral Surg Oral Med Oral Pathol Oral Radiol Endod. 2010;110:691–697. 3. Beirne OR. Evidence to continue oral anticoagulant therapy for ambulatory oral surgery. J Oral Maxillofac Surg. 2005 Apr;63(4):540-5. 4. Broekema FI, van Minnen B, Jansma J, Bos RR. [Risk of bleeding after dentoalveolar surgery in patients taking anticoagulants.] Br J Oral Maxillofac Surg. 2014 Mar;52(3):e15-9 5. Firriolo FJ, Hupp WS. [Beyond warfarin: the new generation of oral anticoagulants and their implications for the management of dental patients.] Oral Surg Oral Med Oral Pathol Oral Radiol. 2012 Apr;113(4):431-41 6. Jeske AH, Suchko GD. ADA Council on Scientific Affairs and Division of Science; Journal of the American Dental Association. [Lack of a scientific basis for routine discontinuation of oral anticoagulation therapy before dental treatment]. J Am Dent Assoc. 2003 Nov;134(11):1492-7 7. Todd DW. Evidence to support an individualized approach to modification of oral anticoagulant therapy for ambulatory oral surgery. J Oral Maxillofac Surg. 2005 Apr;63(4):536-9 8. Wahl MJ. [The mythology of anticoagulation therapy interruption for dental surgery]. J Am Dent Assoc. 2018 Jan;149(1):e1-e10 9. Wahl MJ, et al. [Dental surgery in anticoagulated patients--stop the interruption.] Oral Surg Oral Med Oral Pathol Oral Radiol. 2015 Feb;119(2):136-57 10. Beirne OR, Koehler JR. [Surgical management of patients on warfarin sodium]. J Oral Maxillofac Surg. 1996 Sep;54(9):1115-8 | |
| Randomized clinical trial with control group in use of anticoagulants | 1. Bajkin BV, Popovic SL, Selakovic SD. [Randomized, prospective trial comparing bridging therapy using low-molecular-weight heparin with maintenance of oral anticoagulation during extraction of teeth.] J Oral Maxillofac Surg. 2009 May;67(5):990-5 2. Andrade MVS, Andrade LAP, Bispo AF, Freitas LA, Andrade MQS, Feitosa GS, Feitosa-Filho GS. Evaluation of the Bleeding Intensity of Patients Anticoagulated with Warfarin or Dabigatran Undergoing Dental Procedures. Arq Bras Cardiol. 2018 Sep;111(3):394-399 3. Morimoto Y, Niwa H, Minematsu K. [Risk factors affecting hemorrhage after tooth extraction in patients undergoing continuous infusion with unfractionated heparin]. J Oral Maxillofac Surg. 2012 Mar;70(3):521-6. | |
| Case-control study | 1. Bacci C, Maglione M, Favero L, et al. Management of dental extrac- tion in patients undergoing anticoagulant treatment. Results from a large, multicentre, prospective, case-control study. Thromb Haemost. 2010;104:972–975. 2. Bajkin BV, et al. Dental extractions and risk of bleeding in patients taking single and dual antiplatelettreatment. Br J Oral Maxillofac Surg. 2015 Jan;53(1):39-43 3. Devani P, Lavery KM, Howell CJ. Dental extractions in patients on warfarin: is alteration of anticoagulant regime necessary? Br J Oral Maxillofac Surg. 1998;36:107–111. 4. Miclotte I, Vanhaverbeke M, Agbaje JO, Legrand P, Vanassche T, Verhamme P, Politis C. Pragmatic approach to manage new oral anticoagulants in patients undergoing dental extractions: a prospective case-control study. Clin Oral Investig. 2017 Sep;21(7):2183-2188 5. Zanon E, Martinelli F, Bacci C, Cordioli G, Girolami A. Safety of dental extraction among consecutive patients on oral anticoagulant treatment managed using a specific dental management protocol. Blood Coagul Fibrinolysis. 2003;14(1):27-30. 6. Caliskan M, Tükel HC, Benlidayi ME, Deniz A. [Is it necessary to alter anticoagulation therapy for tooth extraction in patients taking direct oral anticoagulants?] Med Oral Patol Oral Cir Bucal. 2017 Nov 1;22(6):e767-e773 | |
| Study with over 20% of patients using two or more antithrombotic agents or different risks of bleeding | | 1. Cocero N, Mozzati M, Ambrogio M, Bisi M, Morello M, Bergamasco L. [Bleeding rate during oral surgery of oral anticoagulant therapy patients with associated systemic pathologic entities: a prospective study of more than 500 extractions.] J Oral Maxillofac Surg. 2014;72:858–867. 2. Clemm R, et al. [Management of anticoagulated patients in implant therapy: a clinical comparative study.] Clin Oral Implants Res. 2016 Oct;27(10):1274-1282. 3. Olmos-Carrasco O, Pastor-Ramos V, Espinilla-Blanco R, Ortiz-Zárate A, García-Avila I, Rodríguez-Alonso E, et al. [Hemorrhagic complications of dental extractions in 181 patients undergoing double antiplatelet therapy.] J Oral Maxillofac Surg. 2015 Feb;73(2):203-10. 4. Souto JC, Oliver A, Zuazu-Jausoro I, Vives A, Fontcuberta J. [Oral surgery in anticoagulated patients without reducing the dose of oral anticoagulant: a prospective randomized study]. J Oral Maxillofac Surg. 1996 Jan;54(1):27-32 |
| INR out of the interval between 2.0 and 3.0 | | 1. Zanon E, Martinelli F, Bacci C, Cordioli G, Girolami A. [Safety of dental extraction among consecutive patients on oral anticoagulant treatment managed using a specific dental management protocol]. Blood Coagul Fibrinolysis. 2003;14(1):27-30. 2. Sacco R, Sacco M, Carpenedo M, Mannucci PM. [Oral surgery in patients on oral anticoagulant therapy: a randomized comparison of different intensity targets.] Oral Surg Oral Med Oral Pathol Oral Radiol Endod. 2007 Jul;104(1):e18-21 3. Lillis T, Ziakas A, Koskinas K, Tsirlis A, Giannoglou G. [Safety of dental extractions during uninterrupted single or dual antiplatelet treatment]. Am J Cardiol. 2011 Oct 1;108(7):964-7Kumar et al. 2016 4. Devani P, Lavery KM, Howell CJ. [Dental extractions in patients on warfarin: is alteration of anticoagulant regime necessary?] Br J Oral Maxillofac Surg. 1998;36:107–111. 5. Bodner L, Weinstein JM, Baumgarten AK. [Efficacy of fibrin sealant in patients on various levels of oral anticoagulant undergoing oral surgery]. Oral Surg Oral Med Oral Pathol Oral Radiol Endod. 1998;86(4):421-424. 6. Bajkin BV, et al. [Risk factors for bleeding after oral surgery in patients who continued using oralanticoagulant therapy]. J Am Dent Assoc. 2015 Jun;146(6):375-81 7. Iwabuchi H, Imai Y, Asanami S, Shirakawa M, Yamane GY, Ogiuchi H, et al. [Evaluation of postextraction bleeding incidence to compare patients receiving and not receiving warfarin therapy: a cross-sectional, multicentre, observational study]. BMJ Open. 2014 Dec 15;4(12):e005777 8. Febbo A, et al. [Postoperative Bleeding Following Dental Extractions in Patients Anticoagulated With Warfarin. J Oral Maxillofac Surg]. 2016 Aug;74(8):1518-23. 9. Cannon PD, Dharmar VT. Minor oral surgical procedures in patients on oral anticoagulants-a controlled study. Aust Dent J. 2003 Jun;48(2):115-8 10. Bajkin BV, Bajkin IA, Petrovic BB. [The effects of combined oral anticoagulant-aspirin therapy in patients undergoing toothextractions: a prospective study]. J Am Dent Assoc. 2012 Jul;143(7):771-6. 11. Jimson S, et al. [Assessment of bleeding during minor oral surgical procedures and extraction in patients on anticoagulant therapy]. J Pharm Bioallied Sci. 2015 Apr;7(Suppl 1):S134-7 12. Abdullah WA, Khalil H. [Dental extraction in patients on warfarin treatment]. Clin Cosmet Investig Dent. 2014 Aug 19;6:65-9. |
| Letter to the editor | | 1. Curto A. The new oral anticoagulants in oral and maxillofacial surgery. J Oral Maxillofac Surg. 2015 Jun;73(6):1023. 2. Lalla RV, Peterson DE, Aframian DJ. Should warfarin be discontinued before a dental extraction? Oral Surg Oral Med Oral Pathol Oral Radiol. 2012;113:149–150 3. Wahl MJ, Pinto A, Lalla RV. Interruption of warfarin anticoagulation for dental surgery. *Chest*. 2013;144:1424. |
| Study that compared hemostatic modalities | | 1. Sindet-Pedersen S, Ramstrom G, Bernvil S, et al. [Haemostatic effect of tranexamic acid mouthrinse in anticoagulant-treated patients undergoing oral surgery.] N Engl J Med 320:840, 1989 2. Carter G, Goss AN, Lloyd J, Tocchetti R. [Current concepts of the management of dental extractions for patients taking warfarin.] Aust Dent J. 2003;48:89–96. 3. Bublitz R, Sommer S, Weingart D, Bäuerle K, Both A. [Hämostyptische Wundversorgung bei Marcumarpatienten]. Mund Kiefer GesichtsChir (2000) 4 : 240–244 4. Blinder D, Manor Y, Martinowitz U, et al. [Dental extractions in patients maintained on continued oral anticoagulant.] Oral Surg Oral Med Oral Pathol Oral Radiol Endod 88:137, 1999. 5. Halfpenny W, Fraser JS, Adlam DM. [Comparison of 2 hemostatic agents for the prevention of postextraction hemorrhage in patients on anticoagulants]. Oral Surg Oral Med Oral Pathol Oral Radiol Endod. 2001 Sep;92(3):257-9 6. [Bajkin BV](https://www.ncbi.nlm.nih.gov/pubmed/?term=Bajkin%20BV%5BAuthor%5D&cauthor=true&cauthor_uid=25638996), et al. [Comparison of efficacy of local hemostatic modalities in anticoagulated patients undergoing tooth extractions.] Vojnosanit Pregl. 2014 Dec;71(12):1097-101 |
| Studies in which the population is not clearly representative | | 1. Cannon PD, Dharmar VT. Minor oral surgical procedures in patients on oral anticoagulants--a controlled study. Aust Dent J. 2003 Jun;48(2):115-8. |
